# Taking a Fresh Look at Foreign Language Enjoyment Research in SLA: Current Status, Future Directions, and Pedagogical Implications

**DOI:** 10.3389/fpsyg.2021.820025

**Published:** 2022-01-10

**Authors:** Yunxian Guo, Yexiang Qiu

**Affiliations:** ^1^School of College English Teaching and Research, Henan University, Kaifeng, China; ^2^Postdoctoral Research Center for Chinese Language and Literature Studies, Henan University, Kaifeng, China; ^3^School of Chinese Language and Literature, Henan University, Kaifeng, China

**Keywords:** foreign language enjoyment, current status, future direction, pedagogical implication, SLA

## Abstract

Foreign Language Enjoyment (FLE), as the most prevalent positive emotion predicting L2 learners' academic performance and well-being, and a critical factor contributing to the creation of positive micro-institutions (e.g., the classrooms), has received remarkable scholarly attention across the globe in the past eight years. Despite the fact that FLE is the most extensively investigated positive emotion and extant research has yielded rich and invaluable findings, it is far from being adequately studied, leaving vast lacunas to be explored. Therefore, this conceptual review article is written to familiarize language education researchers, practitioners, instructors, and learners with the current status of FLE research and its potential applications in L2 education, and suggest potential avenues for future research. To this aim, by making a diachronical and synchronical delineation of extant literature with regard to the conceptualization and theorization of FLE, and the methodology of FLE research, we argue that it is incumbent on researchers to make a new line of enquiry into the actualization of the ascertained affordances of FLE and its transmission in the microsystem of the classroom. Subsequently, by drawing on the broaden-and-build theory and the control-value theory, we highlight the significance of conducting FLE research with theoretical triangulation and methodological diversity to validate the data and minimum bias. Next, while highlighting the critical role of FLE in L2 education, we suggest some pedagogical implications with the hope of enlightening the practice of key stakeholders such as instructors, teacher educators, and teacher recruiters. In the end, the limitations of existing literature are explicated, and avenues for future studies on FLE in L2 education domain are put forward for interested researchers.

## Introduction

The beginning of the new millennium has witnessed a positive turn of psychology paradigm. Different from traditional psychology whose primary occupation is pathological treatment for abnormalities and disorders people suffer, positive psychology (hereafter PP) appreciates the dialectic nature of well-being, identifies human strengths and virtues that make life good and orients toward the empirical study of how people thrive and flourish (Seligman and Csikszentmihalyi, 2000; Peterson, 2006; MacIntyre and Mercer, 2014). From its inception, PP has been founded on three pillars: (1) positive experiences, (2) positive character traits, and (3) positive institutions. Since MacIntyre and Gregersen (2012) introduced PP to the domain of second language acquisition (hereafter SLA), new knowledge is already being produced across the three topic areas (MacIntyre, 2021).

Most notably, the seminal work of PP has helped shape and strengthen the “affective turn” of SLA research in the past decade (Pavlenko, 2013; Prior, 2019), and underpined the “third phase” of emotion studies in the field emerging in the early 2010s (Dewaele and Li, 2020). Under this novel emotion wave, researchers extended their scholarly attention from an exclusive preoccupation on negative emotions involved in L2 learning and teaching, notably foreign language classroom anxiety (FLCA), to encompass investigations of positive experiences contributing to language learners' well-being and academic performance (Seligman, 2011). Among the wide spectrum of positive emotions L2 learners encounter, enjoyment, is the most frequently experienced and best studied affect (Frenzel et al., 2018; Piniel and Albert, 2018; Dewaele and Li, 2020) since Dewaele and MacIntyre (2014) first introduced the notion of Foreign Language Enjoyment (hereafter FLE) to the field.

The twin frameworks establishing the theorizing triangulation for emotions, particularly for positive emotions, are the broaden-and-build theory (Fredrickson, 2001, 2013) and control-value theory (Pekrun, 2006; Pekrun and Perry, 2014). The broaden-and-build theory proposes that positive emotions—including joy, interest, contentment, pride, and love, share the ability to “broaden people's momentary thought-action repertoires and build their enduring personal resources, ranging from physical and intellectual resources to social and psychological resources” (Fredrickson, 2001, p. 219). The theory also argues that positive emotions perform the function of reducing or undoing the undesirable effects of negative emotions (Fredrickson, 2013). The control-value theory adopts a three-dimensional taxonomy to classify achievement emotions, namely, object focus (activity or outcome emotions), valence (positive or negative), and activation (activating vs. deactivating). It proposes that subjective control and value appraisals are the proximal determinants of achievement emotions while predicting that achievement emotions exert effects on academic performance via their interplay with students' interest and motivation to learn, use of learning strategies, and styles of regulation of learning (see Pekrun, 2006; Pekrun and Perry, 2014; Shao et al., 2019). The theory also posits that, emotions, be it positive or negative, their individual and social antecedents, and their effects constitutes feedback loops, linked by reciprocal causation over time (Pekrun, 2006; Pekrun and Perry, 2014).

FLE, as the most prevailing and pivotal positive emotion predicting L2 learners' academic performance and well-being, and a critical factor contributing to the creation of positive micro-institutions, has drawn considerable attention from researchers across the globe within a decade. Yet, this emotion is far from being fully studied. Existing studies on FLE in SLA domain have yielded rich and invaluable findings concerning the complex, dynamic and transmitting nature, and the wide scope of predictors of this construct, as well as its interaction with other negative emotions, notably FLCA, desirable academic outcomes, and positive personality traits. However, there is no denying that multiple limitations with regard to the scope, theorizing and methodology of FLE research do emerge from extant literature, leaving a vast avenue calling for future exploration. To connect present studies to future researches and the practice of key stakeholders in L2 education, the aim of this paper is to make a delineation of extant literature with ultimate aims of offering a critical overview, illustrating pedagogical implications, and bringing forward future directions of FLE research.

## Foreign Language Enjoyment Research in SLA

### Conceptualizing Foreign Language Enjoyment

Existing literature has proved FLE a rich and multifaceted concept (Dewaele and MacIntyre, 2016). It was properly introduced into the field of SLA by Dewaele and MacIntyre (2014) despite the fact that a few researchers (Green, 1993; Brantmeier, 2005) had paid loose attention to the feeling of enjoyment involved in L2 learning decades ago. Dewaele and MacIntyre (2014) pioneered the study of FLE alongside FLCA, using an online questionnaire to collect a large sample of quantitative and qualitative data from L2 learners around the world, and found that FLE and FLCA were two different dimensions in modest negative correlation rather than the two sides of a coin. They also developed the Foreign Language Enjoyment Scale in that paper, based on Likert scale ratings of 21 items, which has become the main instrument used to measure FLE. Later on, Dewaele and MacIntyre (2016) advanced their exploration of FLE by confirming it an independent dimension from FLCA and conceptualizing it as a “complex emotion, capturing interacting dimensions of challenge and perceived ability that reflect the human drive for success in the face of difficult tasks” (p. 216). They further identified the two sub-categories of FLE, namely FLE-social, defined by “positive feelings, encouraging peers, nice teachers, and a supportive environment” (Dewaele and MacIntyre, 2016, p. 225), and FLE-private, defined as “thoughts and feelings coalescing around a sense of accomplishment” (p. 228). In the same vein, Boudreau et al. (2018) also pinpointed that enjoyment, compared to pleasure, “takes on additional dimensions such as an intellectual focus, heightened attention, and optimal challenge” (p. 153).

The seminal studies of Dewaele and MacIntyre have been the cornerstones of the ensuing flourishing FLE research across the globe. In < one decade, studies on this construct have expanded significantly, gradually crystallizing the conceptualization of FLE. Major findings from existing literature fall into three thematic occupations. First of all, studies on the mechanisms of FLE suggested that it was a dynamic system undergoing fluctuations either on a moment-to-moment time scale or over a much longer period of time, for instance, a semester, owing to both inter-individual and intra-individual variables (Dewaele and MacIntyre, 2016; Dewaele et al., 2016; Dewaele and Dewaele, 2017; Elahi Shirvan and Talebzadeh, 2017; Boudreau et al., 2018; Elahi Shirvan and Taherian, 2018; Elahi Shirvan et al., 2020, 2021a,b). At the micro level, a few researchers found that both subdomains of private-FLE and social-FLE increased longitudinally (Taherian et al., 2021). Learner-internal factors such as motivation, changing attitude to L2 learning, and learner-external variables such as teachers' supportiveness and selection of conversational topics were all reported to affect the formation of FLE (Elahi Shirvan and Talebzadeh, 2017; Teimouri, 2017; Boudreau et al., 2018; Saito et al., 2018; Elahi Shirvan et al., 2021b). Also, FLE was found by some researchers to be a transmissive emotion and enjoyment contagion might take place in teacher-student interactions by automatic mimicry of facial expressions, gestures, and postures like laughter, vocalic expressions, smiling, nodding, and leaning forward (Talebzadeh et al., 2019).

Second, a large bulk of present studies pointed that FLE was a malleable construct highly subject to both learner-internal and teacher/classroom-specific factors (See Dewaele et al., 2018; Li et al., 2018; Jiang and Dewaele, 2019; Mierzwa, 2019; Dewaele and Dewaele, 2020; Elahi Shirvan et al., 2021a; Guo, 2021). To start with, teachers' professional and interpersonal communication skills were found to play a vital role in boosting students' FLE in various contexts (e.g., Dewaele and Alfawzan, 2018; Dewaele and Dewaele, 2020; Guo, 2021; Wang et al., 2021; Xie and Derakhshan, 2021). In addition, teachers' personality traits such as openness, extroversion, and agreeableness were also discovered to be significantly, positively associated with learners' FLE while their conscientiousness and neuroticism did not have significantly similar effects (Ahmadi-Azad et al., 2020). Moreover, learners' personal goals (Mierzwa, 2019; Elahi Shirvan and Talebzadeh, 2020; Guo, 2021), trait emotional intelligence (Li, 2019), grit (Wei et al., 2019; Elahi Shirvan et al., 2021a;), and L2 proficiency (Piechurska-Kuciel, 2017; Li et al., 2020a,b; Guo, 2021), were all found to be significantly correlated with FLE. Additionally, a few researchers also pointed out that some of learners' intellectual humility domains were also negatively associated with FLE (Moskowitz and Dewaele, 2020). Elsewhere, target language was also found a factor to affect learners' FLE in that bilinguals had higher levels of FLE than their monolingual counterparts (De Smet et al., 2018).

Third, FLE, as a positive activating achievement emotion (Pekrun, 2006), was well-claimed to be positively associated with, or have a mediating effect on other desirable academic outcomes or personality traits. Previous studies revealed that there was a significant, positive relationship between FLE and students' self-reported language proficiency and/or their actual academic achievement (Dewaele and Alfawzan, 2018; Jin and Zhang, 2018; Li, 2019; Li et al., 2020b; Guo, 2021). Additionally, higher levels of FLE were reported to be correlated with more willingness to communicate in foreign language classroom (Dewaele and Dewaele, 2018), boosted or more sustainable learner engagement in the language learning process (Jin and Zhang, 2019; Dewaele and Li, 2020; Mercer and Dörnyei, 2020; Guo, 2021), increased levels of L2 grit (Elahi Shirvan et al., 2021a), and better perception of the value of written corrective feedback (Zhang et al., 2021). Moreover, existing literature also indicated that FLE could have an mediating effect on learners' trait emotional intelligence (Li, 2019), and grit (Wei et al., 2019), thus influencing their self-reported and/or actual academic performance ultimately.

In conclusion, previous studies have enlightened the academia with rich and valuable findings concerning the multifaceted, fluctuating and transmitting nature of FLE, its various antecedents, and to some extent its effects on eliciting desirable academic performance and reducing negative experiences. Whereas, it is worth noting that scholarly attention to FLE has been unevenly distributed. For instance, with large bulk of research interest directed to the inquiry of the nature and source of this emotion, the other pole, its actualization and transmission, has been far less explored. Yet, this line of research is equally, if not more, significant given that one of the ultimate goals of language education research is to serve educational practice by improving the effectiveness of teaching and learning.

### Theorizing Foreign Language Enjoyment

As can been seen from the literature above, FLE, as a pivotal positive emotion affecting the effectiveness of L2 teaching and learning, has been extensively studied in the field of SLA. However, present studies of FLE were far from being adequately theorized. Up to now, only a very small proportion of them have been conducted applying the aforementioned frameworks: the broaden-and-build theory (Fredrickson, 2001, 2013) or the control-value theory (Pekrun, 2006; Pekrun and Perry, 2014). Most of such studies still rested content with the appeal for the application of the broaden-and-build theory to the examination of positive emotions, notably FLE, considering that they were able to broaden learners' perspectives, facilitate their resource building and engagement with the language, play, and exploration within unfamiliar settings (MacIntyre and Gregersen, 2012; Dewaele and MacIntyre, 2014; Boudreau et al., 2018), and help learners accumulate social capital (Gregersen et al., 2016), and that that they serve a preventative or protective function against negative emotions (MacIntyre, 2017). Others were calling for a potential integration of the control-value theory with the investigation into academically-related emotions since it addressed both ends of achievement emotions (Li, 2018; Shao et al., 2019; Dewaele and Li, 2020).

However, the repeated appeal for conscious adoptions of either theory to direct academic discussions of FLE has not been well-answered yet. Most researchers tend to proceed with their studies and draw conclusions based on the empirical or experiential data gathered from the participants firsthand. Only very few researchers applied either theory to explore how FLE exerted positive effects on enhancing students' desirable academic performance such as learner engagement (see Guo, 2021), investigate the mechanisms of participants' higher levels of L2 enjoyment than L3 enjoyment, or elucidate the functions and/or determinants of achievement emotions in general from the perspective of the latter framework (Piechurska-Kuciel, 2017; Piniel and Albert, 2018).

### Methodology of Foreign Language Enjoyment Research

Echoing the methodologically emic trend of emotion studies in SLA at the beginning of the 21st century (Dewaele, 2019), FLE research initiated with an overtly extensive adoption of a mixed-methods methodology, embracing both the quantitative and qualitative approaches. Existing mixed-methods studies usually prioritized the quantitative method to collect data and performed statistical analysis with the aim of perceiving the general status of participants' FLE and/or establishing its correlations with other variables such as FLCA, engagement, motivation or personality traits. Accordingly, qualitative approaches collecting participants' perception of FLE experiences were often used as an important supplement for quantitative analyses to gain better insights into the determinants of FLE (see Dewaele and MacIntyre, 2014; Dewaele et al., 2016; Dewaele and Alfawzan, 2018; Li et al., 2018, 2020b; Jiang and Dewaele, 2019; Guo, 2021). Moreover, a couple of other studies deploying mixed-methods approaches distinguished themselves out for their rigor in taking moment-to-moment idiodynamic inroads to unveil the highly unstable relationship between FLE and FLCA (Boudreau et al., 2018), and the dynamic interaction between FLE and different conversational topics (Elahi Shirvan and Talebzadeh, 2017), and disclose the main mechanism of FLE contagion. Idiodynamic approach proves to be more efficient in uncovering the complex nature of FLE and its interaction with negative emotions in the process of L2 learning, yet studies adopting such method are still rare.

However, to take the whole picture of previous FLE studies under examination, it is not difficult to find a dominant relying on quantitative approaches. A large bulk of studies resorted to static cross-sectional designs to establish causal relationship or draw causal inferences between FLE and a wide spectrum of teacher and learner-related variables (see Dewaele and Dewaele, 2017, 2020; Ahmadi-Azad et al., 2020), such as foreign language proficiency (Piechurska-Kuciel, 2017), language achievement (Jin and Zhang, 2018; Li, 2019), personality traits like grit (Wei et al., 2019), classroom environment and trait emotional intelligence (Li, 2019; Li et al., 2020a), and intellectual humility (Moskowitz and Dewaele, 2020). Additionally, an increasing proportion of longitudinal designs of multiple time scales were also used to deepen our understanding of the dynamic and complex nature of FLE including its sub-domains, and/or its relationship with other variables such as FLCA (Dewaele and Dewaele, 2017; Elahi Shirvan and Taherian, 2018; Taherian et al., 2021), motivational factors (Pan and Zhang, 2021), L2 Grit (Elahi Shirvan et al., 2021a), and learners' changing attitude toward L2 learning and the supportive role of the teacher (Elahi Shirvan et al., 2021b).

Given the socially constructed nature of reality (Denzin and Lincoln, 2011), and the highly subjective and fleeting nature of emotions (Dewaele and Li, 2020), qualitative inquiries are indispensable to the investigation of FLE. Nevertheless, pure qualitative studies are rather rare. Current literature shows that only Elahi Shirvan and Taherian (2020) and Elahi Shirvan and Talebzadeh (2020) used purely qualitative method (in-depth interviews) to explore the actualization of potential affordances for FLE in a university course of listening and speaking (the former), or the signature dynamics of FLE and FLCA (the latter), finding that the prototype contributors of FLE were the influence of the teacher and personal goals.

## Discussion and Pedagogical Implications

In this review paper, we gave a critical delineation of current FLE studies in SLA regarding (1) the conceptualization, and (2) the theorization of the construct, and (3) the methodology of FLE research. While the complex, dynamic and transmitting nature of FLE was ascertained and a wide range of facilitators of this emotion were excavated, existing literature also revealed that inadequate research attention has been paid to the actualization or the transmission of this positive emotion, or its interaction with other prevailing positive/negative emotions. Moreover, extant FLE studies were far from being properly theorized despite the repeated calls for a conscious adoption of theoretical frameworks such as the broaden-and-build theory, the control-value theory, and the well-being theory of PP. Additionally, embrace a mixed-methods methodology as FLE research did from the outset, qualitative methods were still underused in this domain, and methodological diversity was needed. In this section, we will show how extant FLE research in SLA pedagogically contributes to L2 education concerning its potentially enlightening guidance for different stakeholders in this domain.

To begin with,with the intense growth of scholarly attention to the emotional dimensions of language learning since the first decade of the 21st century, emotion, apart from the cognitive system, has been discovered to be another crucial factor determining the effectiveness of language learning and teaching. Research findings of emotion studies from the PP perspective have further revealed that positive emotions lead to both better academic performance and enhanced learners well-being. Therefore, L2 teachers are encouraged to take an ecological view toward language learning to integrate positive education with language education (Mercer et al., 2018). One practical view is that teachers apply emotion theories and research findings of FLE studies to their instructional practice by identifying the ascertained facilitators of FLE, thus implementing emotional interventions to help students boost their enjoyment experience in L2 learning (Li and Xu, 2019; Elahi Shirvan and Taherian, 2020). To this end, for instance, teachers can establish rapport in the classroom, create positive classroom atmosphere, and devise stimulating classroom activities with balanced challenges, related to students' immediate concerns, allowing them autonomous chances to present themselves, and enhancing interactions (Dewaele et al., 2019; Mercer and Dörnyei, 2020; Derakhshan et al., 2021; Guo, 2021).

The present paper can also serve language teacher educators and trainers as they can equip language teachers with theoretical knowledge such as the well-being theory of PP and emotion theories (the broaden-and-build theory and the control-value theory) in their teacher education programs and workshops to facilitate teachers' emotion intervention in L2 teaching process. Additionally, given the inherently interpersonal and communicative nature of contemporary foreign language learning and teaching, it is desirable that language teacher educators and trainers incorporate emotional intelligence emotional (EI) and social intelligence (SI) training into their teacher education courses to improve teachers' competencies in perceiving and managing students' emotions, notably FLE, to ultimately increase their professional effectiveness and enhance students' L2 attainment and success (Mercer and Gkonou, 2017).

In the same vein, language teacher recruiters can take advantage of the research findings by extending the scope of standards for selecting quality teachers from an overwhelming emphasis on professional and pedagogical expertise to take into consideration teachers' interpersonal communication skills (Xie and Derakhshan, 2021), as well as their awareness and agency capacity of following the leading edges of educational research and applying the research findings in their teaching practice (Elahi Shirvan and Taherian, 2020; Wang et al., 2021). For instance, recruiting committees can target those language teachers who are conscious of the significance of emotion regulations in effective teaching and learning, willing and able to adjust their teaching pedagogies by employing appropriate interpersonal communication skills to elicit positive emotions (e.g., FLE) and create positive institutions (e.g., classrooms).

To sum up, despite the fact that there are many research lacunas to be filled, existing studies on FLE has yielded valuable findings concerning the conceptualization and theorization of this construct, and the methodology of conducting FLE research. More importantly, current research findings can provide invaluable pedagogical implications for the practice of key stakeholders of language education, for instance, L2 teachers, language teacher educators and trainers as well as teacher recruiting committees.

## Limitations and Directions for Future Research

Despite the fruitful findings of current FLE research in SLA, it should be acknowledged that certain limitations did emerge from extant literature and pinpointing them would presumably shed some light on future research. First of all, studies on FLE was far from being adequately theorized. While the broaden-and-build theory, and the control-value theory were indefatigably commended for the exploration of the predictors and effects of FLE, as well as its interplay with other positive and negative emotions, only a very limited number of studies were undertaken with either theoretical vision. Next to none inquiries have been made into FLE from the perspective of the well-being theory of PP. Owing to the complex and multifaceted nature of FLE, to further conceptualize this construct and gain a deeper insight into the mechanisms of its interaction with other positive/negative achievement emotions and FLE transmission, we highly recommend SLA researchers draw on the broaden-and-build theory of general psychology, the control-value theory of educational psychology, and the well-being theory of PP to perform their future studies to validate the data and minimize bias.

Second, the research scope of FLE needs to be further expanded. While most of the extant studies were devoted to the excavation of various antecedents of FLE and its relationship with another prevalent negative emotion, FLCA, considerable research gaps were created for future exploration. First and foremost, no other researchers except Bayat et al. (2020) and Elahi Shirvan and Taherian (2020) made initiative inquiries to explore how L2 instructors are able to perceive the potential affordances of FLE and actualize them, for instance, via the teacher's inherent multimodality in his corrective feedback, to boost learners' enjoyable experience. Additionally, scarce empirical studies have been undertaken to explore the relationship between FLE and other desirable academic outcomes such as motivation, engagement, and willingness to communicate. Additionally, next to none researches have been conducted to ascertain FLE's role in reducing negative emotions prevailing in L2 learning process other than FLCA. Filling the aforementioned gaps would surely be of both theoretical and practical significance. Therefore, it is incumbent on L2 researchers and practitioners to further study how L2 teachers can draw on or cultivate social-emotional intelligence to perceive learners' enjoyment feeling through their verbal or nonverbal cues (Gregersen et al., 2017), then mobilize their agency capacity to utilize the cues and actualize the predictors of FLE. Additionally, it is advisable that future research attention is directed to the study of FLE transmission in L2 classroom. Educational psychologists have demonstrated that academic emotions were linked to their antecedents and effects by reciprocal causation over time (Pekrun and Linnenbrink-Garcia, 2012). However, despite a very few researchers' tentative efforts made in relation to the “emotion contagion” (Moskowitz and Dewaele, 2019; Talebzadeh et al., 2019), research on the reciprocal nature of FLE transmission in L2 teacher-student interactions is still in its infancy. Therefore, it is incumbent on researchers to study the effects of teachers' enjoyment on students' FLE and vice versa, and explore the interaction patterns between them. Only when we find out how teachers can perceive and actualize the antecedents of FLE, how this activating achievement emotion circulates among teachers and students, can the effectiveness of L2 learning and teaching be really improved and the pedagogical significance of FLE research be brought to full play. Moreover, it is desirable that future researchers extend their occupation circumference so that a wider spectrum of positive emotions and negative emotions prevailing in L2 classroom, notably learner engagement and foreign language boredom, could be brought under examination to better explore how FLE plays its part in enhancing desirable academic performance and alleviating learners' negative experience. Studies of this denomination would presumably yield valuable findings conducive to confirming, even re-conceptualizing the impact of positive emotions mentioned in the broaden-and-build theory or the control-value theory, and providing insightful reference to L2 teaching.

Third, despite the fact FLE research in SLA was performed with a mixed-method methodology from the outset, empirical studies on this topic have been largely quantitative, notably using close-ended questionnaires to elicit participants' static, temporal, and retrospective perceptions and attitudes of FLE with regard to other variables. Though idiodynamic approaches and longitudinal designs of various time-scales employed in some studies have showed their vigor in exploring the dynamic nature of FLE, they are still rarely adopted, let alone pure qualitative methods. When it comes to emotion studies, quantitative and qualitative research are like the left and right eyes of researchers, giving them binocular visions and “allowing them to perceive three-dimensional images of phenomena” (Dewaele, 2019, p. 85). Therefore, future researchers are advised to proceed FLE studies with a richer methodological diversity. Taking advantage of advanced statistics in quantitative studies, such as the idiodynamic and longitudinal approaches, would benefit future research. Meanwhile, diversifying research designs to scale up “longitudinal qualitative interview design, qualitative interviews on multiple timescales, qualitative comparative analysis, retrodictive qualitative modeling, and Q-methodology” (MacIntyre, 2016, p. 13) are also needed in future research. Additionally, it is advisable that future researchers “employ different types of triangulation (e.g., data triangulation, investigator triangulation, theory triangulation and methodological triangulation)” to validate data and “minimize bias in the measurement, sampling and procedure” (Dewaele and Li, 2020, p. 45).

In sum, owing to its critical role in facilitating L2 learners' academic attainment and success and the limitations of extant studies, FLE remains a fertile area of research, and future inquiries call for more conscious theoretical triangulation and greater methodological diversity to validate the data and minimize bias. Also, scholarly attention needs to be directed to a wider scope of thematic occupations to cover, for example, the actualization and transmission of FLE in the microsystem of the classroom, to elicit more profound perceptions of this construct and better applications of the research findings to language education practice to improve the effectiveness of L2 teaching and learning.

## Data Availability Statement

The original contributions presented in the study are included in the article/supplementary material, further inquiries can be directed to the corresponding author.

## Author Contributions

All authors listed have made a substantial, direct, and intellectual contribution to the work and approved it for publication.

## Funding

This study was supported by the Philosophy and Social Science Planning Project of Henan Province of China, entitled A Comparative Study of Australian White and Aboriginal Fictions from the Perspective of Cultural Memory Theory (Grant No. 2021BWX006).

## Conflict of Interest

The authors declare that the research was conducted in the absence of any commercial or financial relationships that could be construed as a potential conflict of interest.

## Publisher's Note

All claims expressed in this article are solely those of the authors and do not necessarily represent those of their affiliated organizations, or those of the publisher, the editors and the reviewers. Any product that may be evaluated in this article, or claim that may be made by its manufacturer, is not guaranteed or endorsed by the publisher.
